# Anti-oomycete Activity of Chlorhexidine Gluconate: Molecular Docking and *in vitro* Studies

**DOI:** 10.3389/fvets.2022.909570

**Published:** 2022-06-17

**Authors:** Dimpal Thakuria, Victoria C. Khangembam, Vinita Pant, Raja Aadil Hussain Bhat, Ritesh Shantilal Tandel, Siva C., Amit Pande, Pramod Kumar Pandey

**Affiliations:** ICAR-Directorate of Coldwater Fisheries Research, Bhimtal, India

**Keywords:** *Saprolegnia*, molecular docking, chlorhexidine, anti-oomycete activity, membrane disruption

## Abstract

Saprolegniosis is one of the most catastrophic oomycete diseases of freshwater fish caused by the members of the genus *Saprolegnia*. The disease is responsible for huge economic losses in the aquaculture industry worldwide. Until 2002, *Saprolegnia* infections were effectively controlled by using malachite green. However, the drug has been banned for use in aquaculture due to its harmful effect. Therefore, it has become important to find an alternate and safe anti-oomycete agent that is effective against *Saprolegnia*. In this study, we investigated the anti-oomycete activity of chlorhexidine gluconate (CHG) against *Saprolegnia*. Before *in vitro* evaluation, molecular docking was carried out to explore the binding of CHG with vital proteins of *Saprolegnia*, such as *S. parasitica* host-targeting protein 1 (SpHtp1), plasma membrane ATPase, and TKL protein kinase. *In silico* studies revealed that CHG binds with these proteins *via* hydrogen bonds and hydrophobic interactions. In an *in vitro* study, the minimum inhibitory concentration (MIC) and minimum fungicidal concentration (MFC) of CHG against *S. parasitica* were found to be 50 mg/L. Further, it was tested against *S. australis*, another species of *Saprolegnia*, and the MIC and MFC were found to be 100 and 200 mg/L, respectively. At 500 mg/L of CHG, there was complete inhibition of the radial growth of *Saprolegnia* hyphae. In propidium iodide (PI) uptake assay, CHG treated hyphae had bright red fluorescence of PI indicating the disruption of the cell membrane. The results of the present study indicated that CHG could effectively inhibit *Saprolegnia* and hence can be used for controlling Saprolegniasis in cultured fish.

## Introduction

*Saprolegnia* is one of the most common oomycetes or fungal-like organisms responsible for devastating diseases in fish. These organisms cause huge losses in aquaculture worldwide and are also considered responsible for the decline in populations of wild fish and amphibians (1–5). The host range of these organisms includes various fish species, amphibians, and crustaceans (6–8). The disease caused by this organism, known as Saprolegniasis, is characterized by white or gray cotton-like mycelial growth at the site of infection. The most common sites of infection are the head, fins, and tail but in severe cases, it spreads further into muscles and blood vessels, and ultimately, the infected fish die due to osmoregulatory failure (9–11).

Earlier, *Saprolegnia* infections were controlled by using malachite green, but the drug has been banned from use in aquaculture due to its carcinogenic, mutagenic, and teratogenic properties (12–14). Since then, various chemicals have been trialed for their anti-oomycete activity against *Saprolegnia* infections and found effective but were accompanied by adverse effects. Among the tested chemicals, formalin was effective but reported to be associated with environmental pollution, a health hazard to the handler, and accumulation in fish flesh making it unfit for consumption (14). Similarly, other chemicals, such as hydrogen peroxide, boric acid, and peracetic acid, which demonstrated anti-*Saprolegnia* activity, are reported to produce negative side effects (15–20). Therefore, it is the need of the hour to discover or identify a chemical that is safe and effective against *Saprolegnia*.

In this study, the anti-*Saprolegnia* activity of chlorhexidine gluconate (CHG) was evaluated. It is the gluconate salt form of chlorhexidine. It is a positively charged polybiguanide that can bind to the negatively charged cell membrane of microbes (21, 22). CHG has broad-spectrum antimicrobial activity with rapid action, killing bacteria within 30 s (23, 24). It also has the ability to prevent microbial colonization and the formation of biofilms (25). It exhibits a dose-dependent effect, being bactericidal at high concentrations (26, 27). Apart from bacteria, CHG is also active against enveloped viruses and most fungi (28). It acts by interfering with cell wall integrity, then attacking the cytoplasmic membrane leading to leakage of cell contents and ultimately cell death (23). It is used as an antiseptic for the prevention of surgical wound infection and as a mouthwash in dentistry (29, 30). When used as an antiseptic for skin, CHG was found to have an extremely low potential for dermal reactions (31). Despite its broad-spectrum antimicrobial effect with good safety profile and widespread use, literature on the effect of CHG against *Saprolegnia* is very limited. There is one patent on a powder formulation developed to treat Saprolegniasis which contains only 1–3 parts of chlorhexidine acetate in combination with other chemicals (Patent no. CN104997805B, China). Therefore, the present study was taken up to evaluate the anti-*Saprolegnia* activity through *in silico* and *in vitro* analysis.

## Materials and Methods

### Culture of *Saprolegnia* Species

For evaluation of the anti-oomycete activity of CHG, two species, *S. parasitica* (Accession no. MT912581) and *S. australis* (Accession no. MT912582), isolated from rainbow trout (*Oncorhynchus mykiss*) were used. Both the isolates were cultured on glucose-yeast extract agar (GYA) plates and incubated at 20 ± 1°C. The isolates were maintained by subculturing into new GYA plates after every few days or when the plate has full hyphal growth. Subculture was done by transferring a piece of agar excised from the advancing edges of hyphal growth to the new GYA plate. In all the experiments, 4-day old culture was used to maintain uniformity.

### *In silico* Analysis of the Interaction Between CHG and Vital Proteins of *Saprolegnia*

The multiple threading approach of I-Tasser was used for homology modeling of *S. parasitica* proteins, i.e., Tyrosine kinase-like (TKL) protein kinase and the plasma membrane ATPase (32). The fold-based approach of the pDOMTHREADER allowed to find a template for the *S. parasitica* host targeting protein-1, as reported earlier (33). Using the standalone version of Modeler 9.18, the template with a high score as per pDOMTHREADER was used for homology modeling (34).

Thereafter, the three-dimensional (3D) structures were refined using ModRefiner (https://zhanglab.ccmb.med.umich.edu/ModRefiner/) and the quality was checked by the saves server [SAVESv6.0—Structure Validation Server (ucla.edu)]. In each modeled protein, the binding sites were predicted by the COACH meta server (35). CHG structure was retrieved from Pubchemin in SDF format. The binding energy of ligand-receptor complexes was computed using AutoDockVina software by mimicking the ligand into the active site of the protein (36). There were nine docking poses predicted for each ligand-receptor complex when the exhaustiveness was set to twenty. Among these, the ideal pose of binding between protein and ligand was chosen to explore hydrogen bonding and hydrophobic interactions, with a higher negative docking score (37). PyMOL was used to visualize 3D structures and docking structures, whereas LigPlot 2.1 was used to visualize two-dimensional (2D) structures (38).

### Preparation of Chlorhexidine and Resazurin Stock

A stock solution of 10,000 mg/L of CHG was prepared using sterile double-distilled water. Working solutions were prepared by diluting them with sterile double-distilled water. A stock solution of resazurin dye was prepared by dissolving 25 mg in 10 ml of 10 mM sodium phosphate buffer (pH, 7.4). The solution was then vortexed till the dye dissolves completely and sterile filtered using a 0.2 μm syringe filter and stored at −20°C until use. A working solution of resazurin (300 μM) in sodium phosphate buffer was prepared fresh before each experiment.

### Determination of Minimum Inhibitory Concentration

The minimum inhibitory concentration (MIC) of CHG was determined against *S. parasitica* and *S. australis*, following the published methodologies (39, 40) with some modifications. In a 48-well plate, 400 μl of glucose yeast extract broth (GYB) containing different concentrations (1, 10, 20, 50, 100, and 200 mg/L) of CHG was added. Triplicate wells of each concentration of CHG were used for the assay. Growth control wells without CHG and sterile control wells containing only media were included in the experiment. In each well except the sterile control wells, an agar plug of approximately 4 mm in diameter derived from a GYA plate containing 4-day old culture of *Saprolegnia* was added. The plate was incubated at 20 ± 1°C for 48 h. Thereafter, 100 μl of freshly prepared resazurin (300 μM) was added to each well. The plate was further incubated and observed for change in color from blue to pink. The minimum concentration of CHG at which the color remained blue was recorded as MIC.

### Determination of Minimum Fungicidal Concentration

For the determination of minimum fungicidal concentration (MFC), the experiment was carried out in a 48-well plate similar to that of MIC. Agar plugs containing *Saprolegnia* hyphae were inoculated in separate wells containing different concentrations of CHG (sub-MIC, MIC, and 2X MIC) and incubated for 24 h at 20 ± 1°C. Growth control wells containing only media and *Saprolegnia* but no CHG were included in the experiment. After incubation, the agar plugs from control and the CHG treated wells were transferred to GYA plates and incubated for 24 h at 20 ± 1°C. The agar plates were observed for the presence or absence of any visible hyphal growth. The minimum concentration of CHG at which there was no visible hyphal growth was recorded as an MFC value. Further, the effect of CHG on live fish was checked by dip treatment at MIC and MFC for a short period of time.

### Radial Growth Inhibition Assay

The effect of CHG on the radial growth of *Saprolegnia* hyphae was determined in a time series following the protocol of Shin et al. (39). In brief, GYA containing different concentrations of CHG (25, 50, 100, 200, 500, and 1,000 mg/L) was prepared by mixing an equal volume of 2X GYA and 2X CHG to achieve the desired final concentration. The GYA containing CHG was then plated on sterile 90 mm Petri dishes. A growth control plate containing 2X GYA mixed with an equal volume of sterile distilled water was included in the experiment. An agar plug of ~5 mm diameter containing *Saprolegnia* hyphae was cut from 4-day old culture plate and placed onto the center of the fresh GYA plate. The plates were incubated at 20 ± 1°C, and radial growth was recorded as diameter every 24 h till full mycelial growth was achieved in the growth control plate. The experiment was conducted in triplicates and repeated three times. The percentage inhibition of radial growth (PIRG) was calculated using the formula, PIRG = (DC–DT) ×100/DC, where, DC = diameter of radial growth on the control plate in mm, and DT = diameter of radial growth on CHG treated plate in mm. The values are presented as mean ± SE.

### Analysis of Membrane Integrity

A propidium iodide (PI) uptake assay was performed to investigate the effect of CHG on membrane integrity of *Saprolegnia* following the protocol of Palem et al. (41) and Shin et al. (39) with some alterations. *Saprolegnia* was inoculated on a GYA plate along with sesame seeds which were used as bait. After 4 days of incubation, the sesame seeds bearing *Saprolegnia* hyphae were transferred to 48 well plates containing GYB incorporated with MIC and MFC concentration of CHG and incubated at 20 ± 1°C. Growth or negative control wells containing only medium and positive control wells containing medium plus 0.1% triton-X were included in the experiment. After incubation for 24 h, the hypha was washed in PBS and stained with PI (20 μg/ml) for 20 min in dark at room temperature. The PI fluorescence was observed under an inverted fluorescent microscope (Nikon, Japan).

## Results

### *In silico* Analysis of the Interaction Between *Saprolegnia* Proteins and CHG

**Figure 1:**
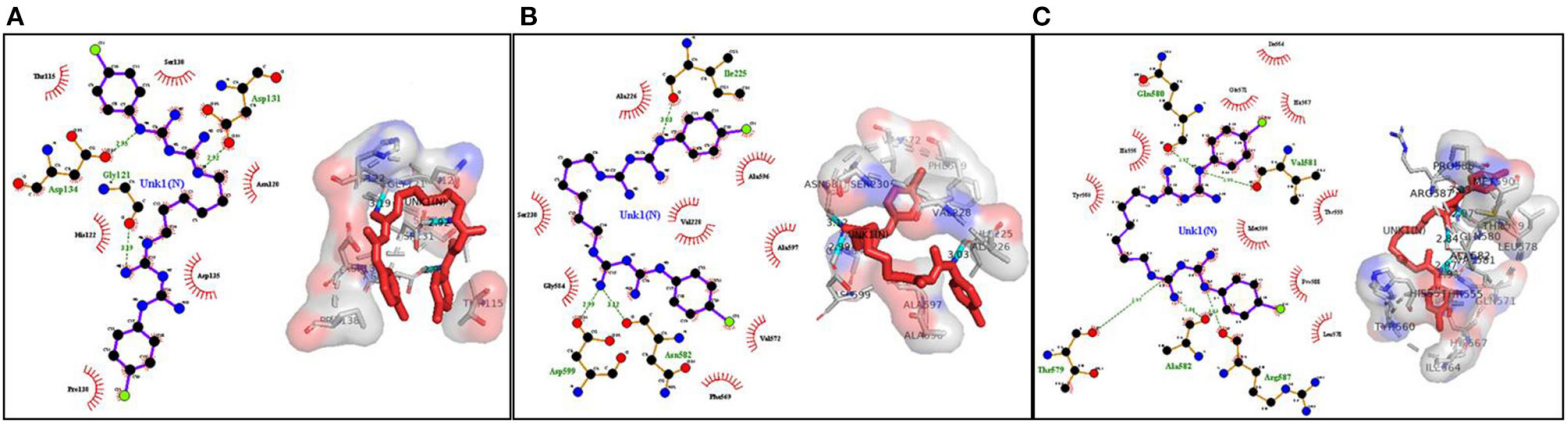
Molecular docking of CHG with proteins of *S. parasitica*. **(A)** with *S. parasitica* host targeting protein, **(B)** with plasma membrane ATPase, **(C)** with TKL protein kinase.

The quality of modeled 3D structure was analyzed through the Ramachandran plot. It was found that about 97% of amino acid residues in the modeled structure were in the most favored region. Details of *in silico* analysis of the 2D and 3D interactions between the active sites of *Saprolegnia* proteins and CHG ligand are given in Figure 1, Table 1. Among the proteins studied through molecular docking, the lowest minimum binding energy, −9.1 Kcal/mol, was observed between CHG and TKL protein kinase. CHG was found to bind strongly with TKL protein kinase through five hydrogen bonds and nine hydrophobic interactions. Similarly, the ligand has shown good interaction with plasma membrane ATPase through three hydrogen bonds and eight hydrophobic interactions, with a binding energy of −7.7 Kcal/mol. CHG also interacted with *S. parasitica* host-targeting protein 1 (SpHtp1) through two hydrogen bonds and six hydrophobic interactions with a binding energy of −6.0 Kcal/mol.

**Table 1:** Details of interactions between *Saprolegnia* proteins and chlorhexidine gluconate (PubChem CID: 9552079).

**Name of the protein**	**Residues involved in hydrophobic interactions**	**Residues involved in hydrogen bonding: bond length (Å)**	**Total no. of hydrogen bond**	**Docking energy (kcal/mol)**
Saprolegnia parasitica host-targeting protein 1 (SpHtp1)	Thr115, Ser130, Asn120, Asp135, His122, pro138	Gly121: 3.19, Asp134: 2.95	2	−6.0
Plasma membrane ATPase	Ser230, Ala22, ala596, Val228, Gly584, ala597, val572, Phe569	Asp599:2.99 Asn582:3.12 Ile225:3.03	3	−7.7
TKL protein kinase	Ile564, Gln571, His567, Thr555, Met590, Pro588, Leu78, His556, Tyr560	Gln580:2.97, Val581:2.99 Thr597:2.97, Ala582:2.4 Arg587:3.03	5	−9.1

### Minimum Inhibitory and Fungicidal Concentration

**Figure 2:**
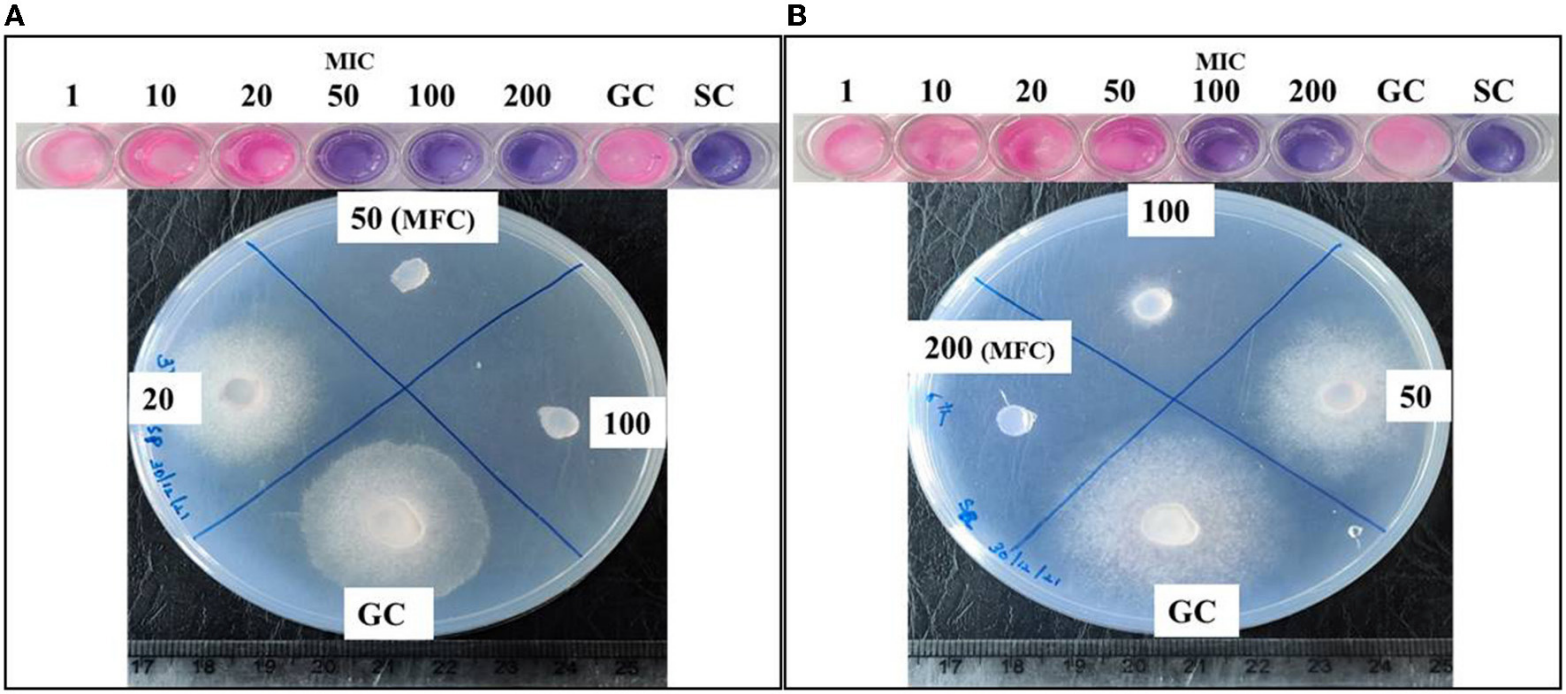
Determination of minimum inhibitory concentration (MIC) and minimum fungicidal concentration (MFC) of CHG against *Saprolegnia*. Series of wells with blue and pink color at the top is of resazurin assay for determination of MIC. **(A)** MIC (blue) and MFC is 50 mg/L for *S parasitica*, **(B)** MIC is 100 mg/L and MFC is 200 mg/L for *S. australis*. Values are concentration of CHG in mg/L. GC, Growth control; SC, Sterile control. In wells of MIC and above, there is no development of pink color. In MFC, hyphal growth is absent.

Chlorhexidine gluconate was found to inhibit the growth of *S. parasitica* and *S. australis* at different concentrations. The minimum concentration of CHG (MIC), which inhibited the growth of *Saprolegnia*, observed as an unchanged blue color of resazurin, was found to be 50 and 100 mg/L for *S. parasitica* and S. *australis*, respectively. In the case of *S. parasitica*, MFC was the same as that of MIC, i.e., 50 mg/L. It was evident from the absence of hyphal growth when the CHG treated specimen was inoculated on a fresh GYA plate. However, the MFC of CHG to produce a fungicidal effect in *S. australis* was found to be 200 mg/L which was two times its MIC value (Figure 2). Fish treated with 100 and 200 mg/L of CHG by dip method showed no abnormal behavior, changes in feed intake, and gross lesions in the skin during the observation of a period of 96 h after treatment.

### Radial Growth Inhibition

**Figure 3:**
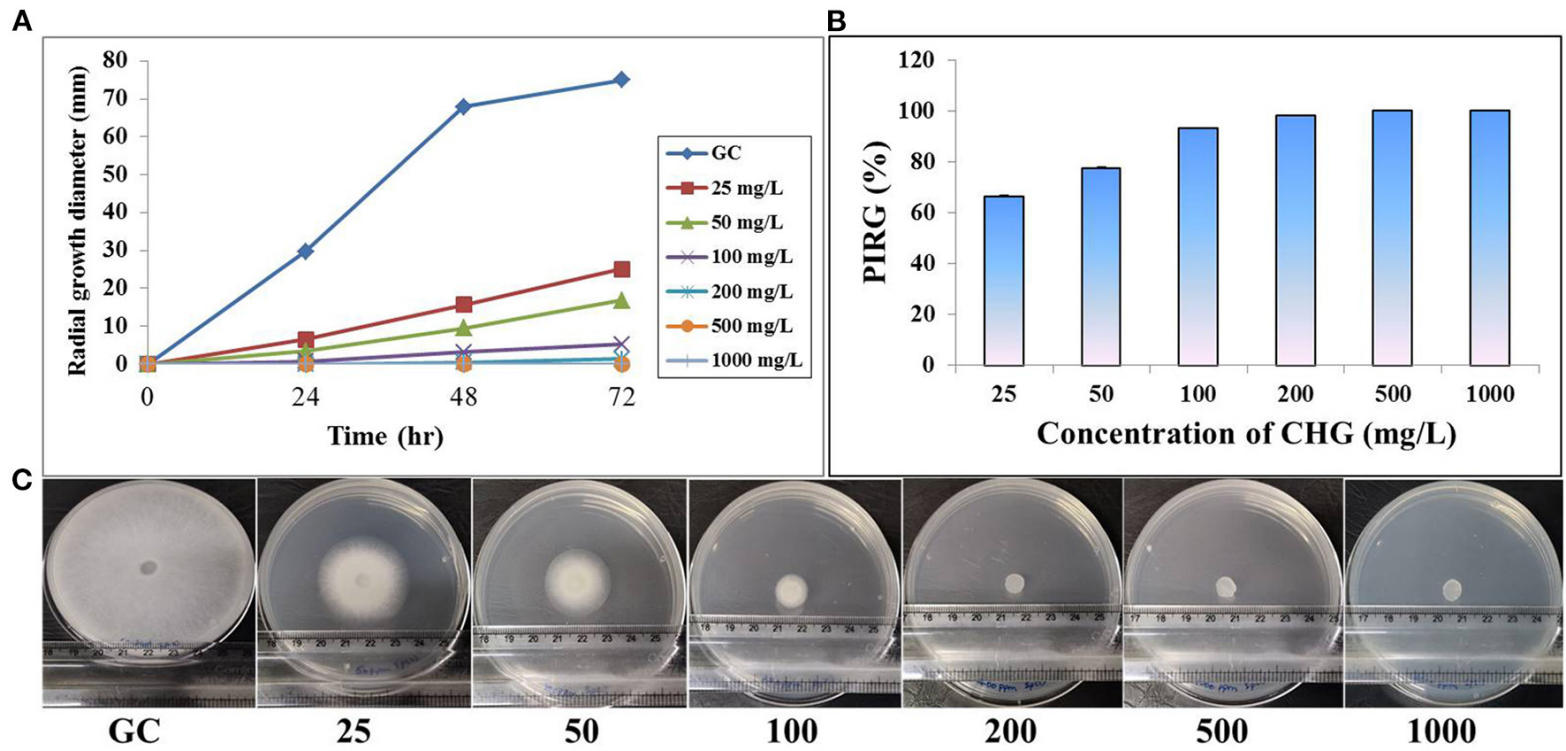
Effect of CHG on radial growth of *Saprolegnia parasitica*. **(A)**Time dependent mycelial growth of *S. parasitica* on glucose yeast extract agar incorporated with different concentrations of chlorhexidine (0, 25, 50, 100, 200, 500 and 1000 mg/L). **(B)** Estimation of percent inhibition on radial growth of *S. parasitica* on CHG incorporated glucose yeast extract at 72 hr. **(C)** Full grown hyphae in growth control (GC) and reduced or absence of hyphal growth in CHG treated plates at 72 hr.

**Figure 4:**
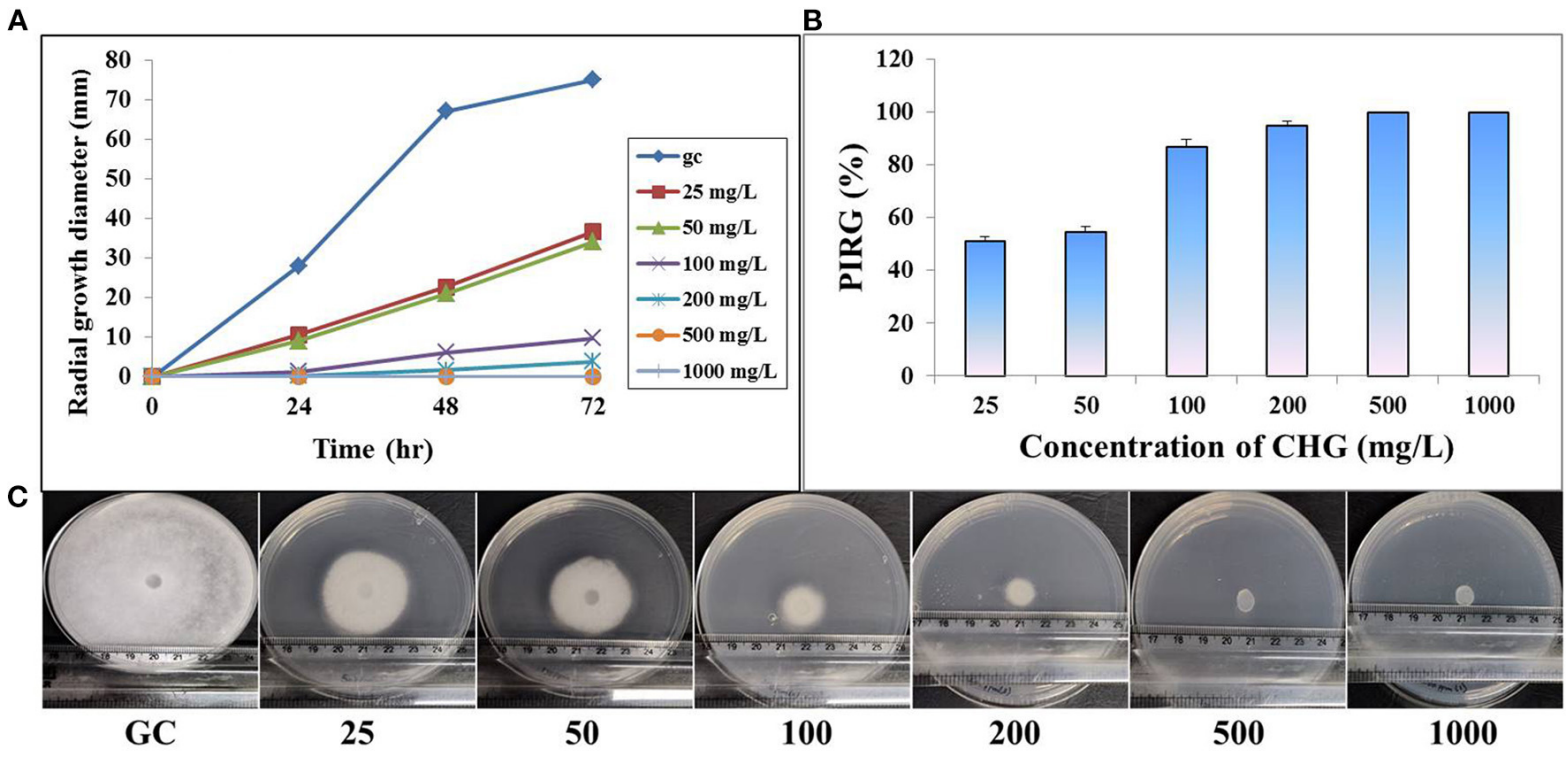
Effect of CHG on radial growth of *Saprolegnia australis*. **(A)** Time dependent mycelial growth of *S. australis* on glucose yeast extract agar incorporated with different concentrations of chlorhexidine (0, 25, 50, 100, 200, 500, and 1,000 mg/L). **(B)** Estimation of percent inhibition on radial growth of *S. australis* on CHG incorporated glucose yeast extract at 72 hr. **(C)** Full grown hyphae in growth control (GC) and reduced or absence of hyphal growth in CHG treated plates at 72 hr.

On CHG supplemented GYA, radial growth of *Saprolegnia* hyphae was inhibited in a dose-dependent manner. The mean diameter of radial hyphal growth decreased with an increased concentration of CHG in the medium. At higher concentrations, 500 and 1,000 mg/L, there was complete inhibition in the radial growth of both the species. At 72 h, the percentage rate of inhibition in radial growth at 25 mg/L was found to be more than 60 and 50% for *S. parasitica* and *S. australis*, respectively (Figures 3, 4).

### Disruption of Membrane Integrity

**Figure 5:**
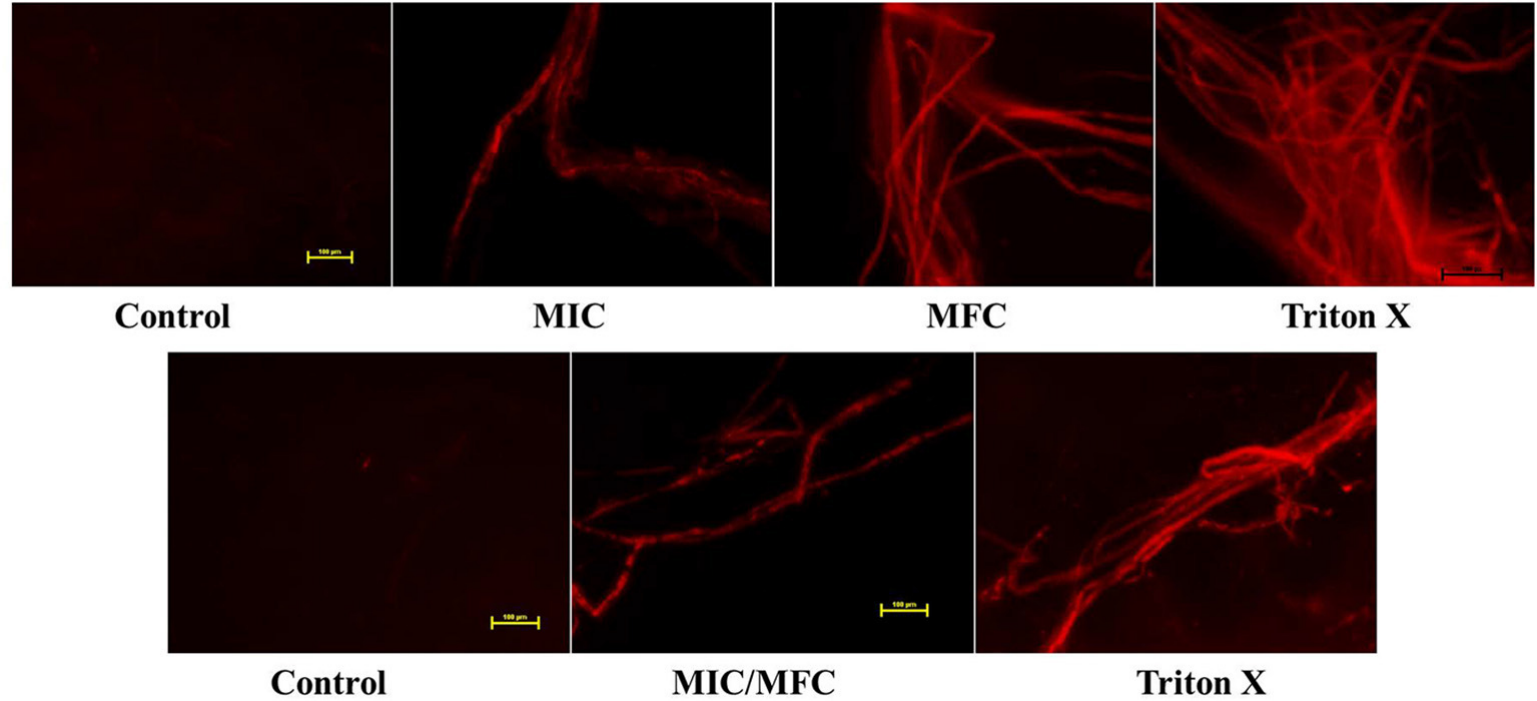
PI uptake assay to investigate membrane disruption by chlorhexidine. The first row is of *S. australis* treated with 0, MIC and MFC of CHG and Triton-X 100. The second row is of *S. parasitica* treated with 0, MIC/MFC of CHG and Triton-X 100. Scale-100 μm.

In the PI uptake assay, Sa*prolegnia* hyphae, treated with MIC and MFC of CHG, showed bright red fluorescence indicating loss of membrane integrity. Red fluorescence of high intensity was also observed in Triton-X treated *Saprolegnia*. The untreated negative control hyphae had no red fluorescence indicating an intact membrane (Figure 5).

## Discussion

*Saprolegnia* species, particularly *S. parasitica*, have re-emerged as an important pathogen in aquaculture due to a lack of effective control measures. After malachite green has been banned for use in aquaculture, several chemicals have been evaluated for their efficacy to control Saprolegniasis. However, the side effects of those chemicals on the handler, fish, and environment have been the main obstacles for field application in aquaculture (20). This has prompted us to search for new chemicals that are effective against *Saprolegnia* and safe to use as well. Thus, the current study was undertaken to evaluate the anti-*Saprolegnia* activity of CHG *in vitro*. Chlorhexidine is commonly known for its salt forms, such as chlorhexidine acetate, CHG, and chlorhexidine digluconate. CHG is one of the most widely used broad-spectrum antiseptics, effective against various microbial pathogens with low toxicity to the host (27, 42–44). Owing to its low potential for toxicity, CHG has been incorporated in many cosmetic products, antiseptic creams used for wound cleaning, and teat dip in the dairy industry. It is one of the most commonly used chemicals in dentistry and as an adjunct in the treatment of oral yeast infections (45, 46).

Before carrying out the *in vitro* studies, the probable interaction of CHG with vital proteins of *S. parasitica* was explored through molecular docking. This bioinformatic approach allows to identification potential inhibitory molecules of the oomycete vital proteins, responsible for virulence (33, 46). Recently, Kumar et al. (20) have also identified several compounds along with *Saprolegnia* target proteins using the computational proteomics approach. In this study, CHG was found to interact with *Saprolegnia* proteins (SpHtp1, TKL kinase, and plasma membrane ATPase) through hydrogen bonds and hydrophobic interactions, which revealed the potential of the anti-*Saprolegnia* activity. SpHtp1 translocates inside fish cells in a tyrosine-O-sulfate–dependent manner and may play a role in saprolegniasis (47, 48). Similarly, TKL protein kinase and plasma membrane ATPase are important proteins that can be targeted for the discovery of antifungal agents (49, 50). The interaction of TKL protein kinase and plasma membrane ATPase with CHG obeyed Lipinski's rule of five (51). It is a rule of thumb to evaluate the drug-likeness of a chemical compound. Based on the findings of molecular docking, CHG was predicted to produce an inhibitory effect against *Saprolegnia*.

The efficacy of CHG was evaluated *in vitro* against two species, *S. parasitica* and *S. australis*. CHG produced its inhibitory and fungicidal effect at 50 mg/L which corresponds to 0.005% against *S. parasitica*. Whereas, higher dosage of CHG, i.e., 100 mg/L or 0.01% for growth inhibition and 200 mg/L 0.02% for the killing of *S. australis* were required. This difference in sensitivity can be corroborated by reports on the variable efficacy of boric acid among *Saprolegnia* species (52). Such variation in antifungal effect against different species has also been reported in *Aspergillus flavus* and *Aspergillus niger* (53). This may be due to differences in the cell wall or membrane component between the species (54). In addition, we have observed such variation in the efficacy of antimicrobials in bacteria among different species of *Aeromonas, i.e., Aeromonas sobria, Aeromonas hydrophilla*, and *Aeromonas salmonicida* (37, 55).

Chlorhexidine gluconate was found to produce its growth inhibitory effect against the two tested species of *Saprolegnia* at 0.005 to 0.01% and its biocidal effect at 0.005 to 0.02%. The effective dose is much lesser than that of common concentrations of CHG used for medicinal purposes. Most widely available chlorhexidine products contain 2% active chlorhexidine in its salt form either gluconate or acetate (56). It was reported that 0.02% chlorhexidine produced no abnormal behavior and no significant changes in hematological and serum biochemical parameters in laboratory rats (57). Wade et al. (58) stated that alcoholic formulations of 4–5% of CHG seem to be safe and effective in preventing infection after clean surgery in adults. This indicates that CHG can produce its growth inhibitory and biocidal effect against *Saprolegnia* at a safe concentration for the handler.

Chlorhexidine gluconate was found to cause complete inhibition of radial growth at ≥500 mg/L in both the species of *Saprolegnia*. It is less than the concentration of boric acid required to produce a similar effect (59). Moreover, the MIC and MFC of CHG are also much lesser than that of boric acid. This may be well explained by the mode of action of chlorhexidine. CHG kills bacteria and fungus through disruption in structural organization and integrity of the cytoplasmic membrane, resulting in leakage of intracellular components (23). As expected, in the PI uptake assay, CHG-treated *Saprolegnia* hypha showed red fluorescence but the un-treated one did not. This suggested that CHG disrupted the cell membrane and allowed the membrane impermeant dye, PI to enter inside the cells, and stained nucleic acid. As per the literature, chlorhexidine can also kill the microbes within a very less time of contact (57) making it suitable for dip treatment. In fish, dip treatments are done by keeping the fish in a strong solution of chemicals for <1 min (60). We also found no gross changes in the fish treated with MIC and MFC of CHG.

With our findings, it is confirmed that CHG can bind strongly with important proteins of *Saprolegnia*, which depicts its anti-oomycete potential. The inhibitory effect of CHG against *Saprolegnia* was further demonstrated through various *in vitro* assays. It produced its inhibitory and fungicidal effect against *Saprolegnia* species at the sub-therapeutic level. As there is a lack of effective prophylactic and therapeutic measures against *Saprolegnia*, an effective and eco-friendly chemical is highly essential to control Saprolegniasis. Therefore, CHG, with high efficacy against various microbial pathogens and negligible toxicity can be an ideal candidate for application in aquaculture for the management of Saprolegniasis. Further, CHG can be incorporated as a major component in formulations for the treatment of Saprolegniasis. As downstream work, CHG may be evaluated for its efficacy for disinfection of fish eggs and treatment of Saprolegniasis in adult fish.

## Data Availability Statement

The original contributions presented in the study are included in the article/supplementary material, further inquiries can be directed to the corresponding author.

## Ethics Statement

The animal study was reviewed and approved by Institutional Animal Care and Use Committee, ICAR-DCFR.

## Author Contributions

DT and VCK conceptualized and designed the experiments. DT, VCK, and VP conducted the *in vitro* assays and wrote the manuscript. RAHB did *in silico* analysis. AP conducted fluorescent microscopy. RST contributed in culture of *Saprolegnia*. SC, DT, and VCK conducted the dip treatment of fish. DT, VCK, and PKP edited the manuscript.

## Funding

This work was supported by the ICAR Institutional fund under the project “Evaluation of antimicrobial potential of nano and polymer based formulations against saprolegniasis”.

## Conflict of Interest

The authors declare that the research was conducted in the absence of any commercial or financial relationships that could be construed as a potential conflict of interest.

## Publisher's Note

All claims expressed in this article are solely those of the authors and do not necessarily represent those of their affiliated organizations, or those of the publisher, the editors and the reviewers. Any product that may be evaluated in this article, or claim that may be made by its manufacturer, is not guaranteed or endorsed by the publisher.
